# Tissue Nutrient Environments and Their Effect on Regulatory T Cell Biology

**DOI:** 10.3389/fimmu.2021.637960

**Published:** 2021-04-02

**Authors:** Julianna Blagih, Marc Hennequart, Fabio Zani

**Affiliations:** The Francis Crick Institute, London, United Kingdom

**Keywords:** regulatory T cells, metabolism, tissues, metabolic adaptation, nutrients

## Abstract

Regulatory T cells (Tregs) are essential for mitigating inflammation. Tregs are found in nearly every tissue and play either beneficial or harmful roles in the host. The availability of various nutrients can either enhance or impair Treg function. Mitochondrial oxidative metabolism plays a major role in supporting Treg differentiation and fitness. While Tregs rely heavily on oxidation of fatty acids to support mitochondrial activity, they have found ways to adapt to different tissue types, such as tumors, to survive in competitive environments. In addition, metabolic by-products from commensal organisms in the gut also have a profound impact on Treg differentiation. In this review, we will focus on the core metabolic pathways engaged in Tregs, especially in the context of tissue nutrient environments, and how they can affect Treg function, stability and differentiation.

## Introduction

The immune system is in a dynamic balance between inflammation and suppression. One critical population in helping maintain this balance are regulatory T cells (Treg), a population first identified in the mid-nineties (1). Humans bearing mutations in the forkhead box P3 (*FOXP3*) gene, which leads to lethal autoimmunity, helped identify FOXP3 as the signature transcription factor for Treg lineages (2). Tregs are derived from two different origins. Thymic Tregs (tTregs), also known as natural Tregs (nTregs), are selected and educated during thymic development and egress to secondary lymphoid organs (SLO). To further secure against uncontrolled inflammation, Tregs arising from naïve CD4 T cells in the SLO, termed inducible Tregs (iTregs), require cues from IL2 and TGFβ for differentiation (3).

The overarching function of Tregs is to maintain peripheral tolerance, such as in the colon which commensal bacteria live in harmony with the host. Other functions include preventing autoimmunity, limiting inflammation once the infection has been cleared and tissue repair, which all benefit the host (4). However, there are situations where Tregs become harmful, such as in tumors where they tip the balance towards tumor growth (5). There are several methods of suppression that modulate immune responses: cytokine mediated inhibition (i.e. IL10 and TGFβ), IL-2 deprivation, prevention of dendritic cell (DC) maturation, and cytolysis (6–8).

Treg development and suppressive programs depend on signalling downstream of the T cell receptor (TCR), CD28 and the IL2 receptor (CD25). These different inputs work either in tandem or in parallel to regulate FoxP3 expression. TCR engagement induces the activation of Class IA inositide lipid kinases, PI3Ks, to recruit PDK1 and Akt to the plasma membrane. Phosphorylation of Akt by PDK1 maintains suppressive function and response to TCR signal strength in Tregs (9). This signalling axis shapes Treg fate by regulating the FOXO family of transcription factors and the activation of the mammalian target of rapamycin complex I (mTORC1) (10, 11). STAT5, a transcription factor downstream of IL2 signalling, also determines Treg polarization, proliferation, stability and competitive fitness (12–15).

Many of these transduction pathways converge on cellular metabolism. Naïve T cells are in a state of quiescence and their metabolic demand is therefore low; however, once activated T cells upregulate anabolic and catabolic metabolism including glycolysis and amino acid synthesis (16–18). A large number of these anabolic pathways are reliant on adenosine triphosphate (ATP) which is mainly produced from glycolysis and mitochondrial oxidative phosphorylation (OXPHOS). Besides proliferation, defined metabolic changes are required to support specific T cell fates. For example, CD8^+^ effector T cells transitioning into central memory cells rewire their metabolism from glycolysis towards fatty acid oxidation (FAO) (19). Complex metabolic alterations also support the differentiation of CD4^+^ T cells towards different T_helper_ subsets. This topic has been recently reviewed by Shyer et al. and Geltink et al. and will not be the focus of this review (20, 21).

Treg metabolism is a growing area of research in the field of immunometabolism. In this review, we will focus on the metabolic features, metabolites, and metabolic modulators that influence Treg function, homeostasis, and plasticity. While lymphoid organs may perhaps be metabolically rich environments, Tregs extravasating to tissue sites face notably different nutrient availability depending on their location. How different metabolic environments affect Treg cell fate and biology will be further explored in this review.

## Fatty Acids and Mitochondrial Metabolism in Tregs

One key organelle that shapes Treg cell fate and function is the mitochondrion. The metabolic pathways that support mitochondrial activity and cell energy production are the tricarboxylic acid cycle (TCA), amino acid and lipid metabolism.

**Figure 1:**
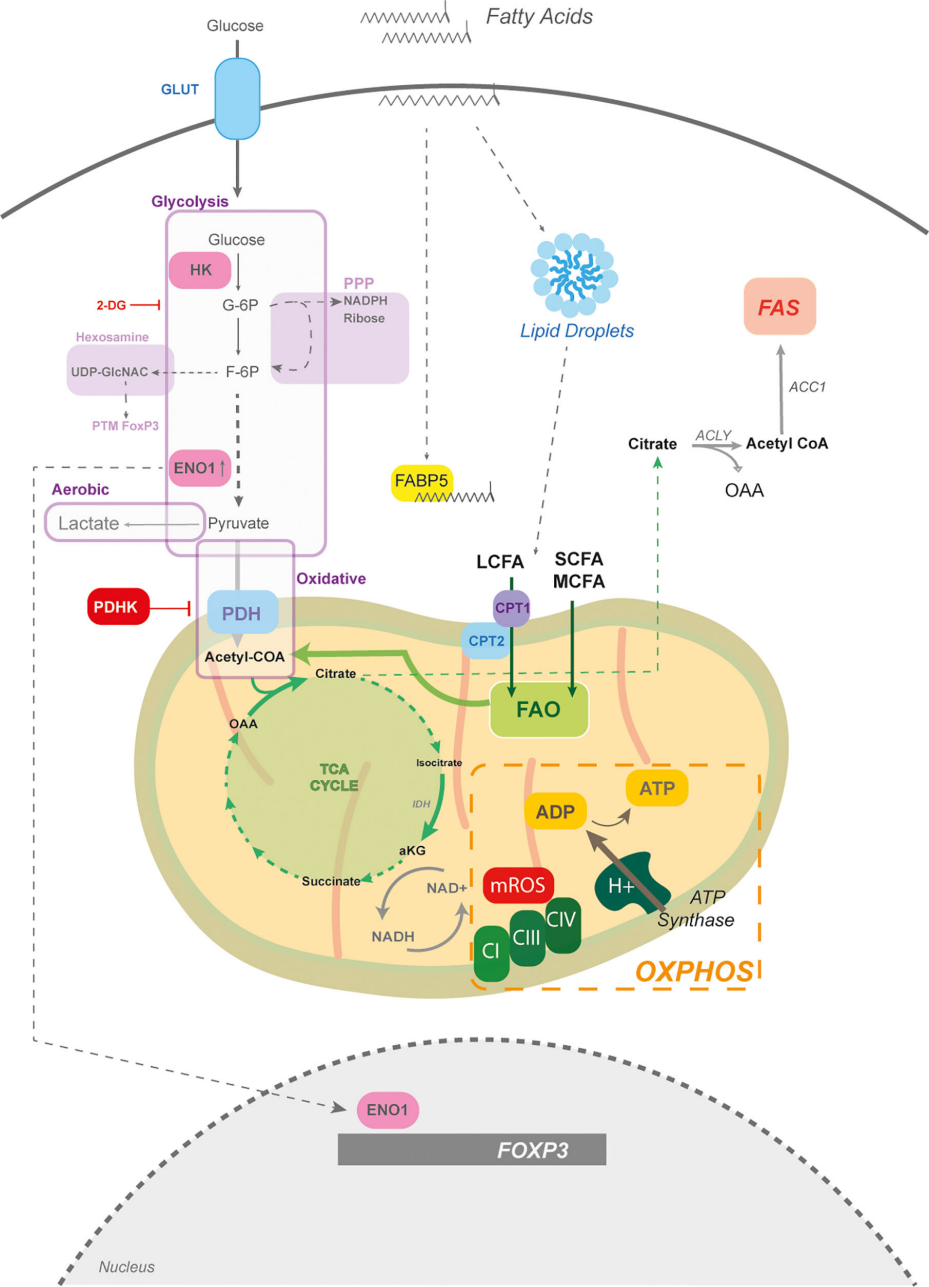
The mitochondria and fatty acid metabolism in Tregs. The core of Treg metabolism centres around mitochondrial activity, which is partially supported by glycolysis. Glycolysis is the breakdown of glucose into pyruvate; however, glycolytic intermediates can feed into other pathways, such as the pentose phosphate pathway (PPP) for NADPH production and the hexosamine pathway for amine sugar production (i.e. UDP-GlcNac) involved in post-translational modifications (PTM). If the end product of glycolysis is pyruvate, which can enter the mitochondria, it is termed oxidative glycolysis. However, if glucose-derived pyruvate is shunted toward lactate production it is termed aerobic glycolysis. Pyruvate enters mitochondria through the pyruvate dehydrogenase (PDH) and generates acetyl CoA to drive the tricarboxylic acid (TCA) cycle. PHD is negatively regulated by PDH kinase. While the TCA cycle can generate amino acids, its function is to regenerate NADH which is critical for electron transport chain (ETC) function. The ETC, composed of complexes I-IV, builds a proton motif force to drive the generation of ATP by ATP synthase. Mitochondrial reactive oxygen species (mROS) are produced at complex I and III. Fatty acids through fatty acid oxidation (FAO) can also provide acetyl CoA units for the TCA cycle. Tregs store fatty acids in the form of lipid droplets, which are partially taken up from the environment. The fatty acid binding protein (FABP5) is a chaperone for long chain fatty acids (LCFAs) and plays a key role in mitochondrial structure. LCFAs require assisted entry into the mitochondria, which is mediated by CPT1 and CPT2. Other sources of fatty acids are the short chain (SCFAs) and medium chain fatty acids (MCFA), that pass freely into the mitochondria. Tregs also perform fatty acid synthesis (FAS) which takes mitochondrial citrate and exports it into the cytoplasm to regenerate acetyl CoA by ATP citrate lyase (ACLY). Acetyl CoA is then committed to fatty acid synthesis by the acetyl CoA carboxylase (ACC). The glycolytic enzyme, enolase (ENO1) can also moonlight as a transcription factor and bind to the *FOXP3* promoter to mediate the generation of different splice forms.

A major source of ATP is derived from mitochondrial oxidative phosphorylation (OXPHOS) which is driven by the electron transport chain (ETC). The ETC and TCA cycle are intrinsically coupled processes. For example, NADH/NAD^+^ recycling in the mitochondria relies on the TCA cycle regenerating NADH, an electron donor, for the ETC (22). The ETC comprises four subunits (Complex I-IV) and an ATP synthase (**Figure 1**). Treg-specific deletion of ETC components remarkably impair Treg suppressive function, underpinning a fundamental function for mitochondrial OXPHOS in Tregs (23–25). The TCA cycle is driven by the oxidation of Acetyl CoA, which could be generated from glucose-derived pyruvate through the pyruvate dehydrogenase (PDH) complex (**Figure 1**). Deletion of the negative regulator of PDH (PDH kinase) increases PDH activity thus favoring oxidative cellular metabolism for iTreg differentiation (26).

Genetic perturbations in regulators involved in maintaining mitochondrial oxidative activity, such as the mitochondrial transcription factor TFAM, reduce Treg differentiation, function and result in lethal systemic autoimmunity (25, 27–29). Other sources of acetyl CoA that can enter the TCA cycle come from fatty acid oxidation (FAO), which catabolizes fatty acids within the mitochondria into acetyl CoA units. Tregs take up fatty acids and store them in the form of lipid droplets (30, 31) (**Figure 1**). Surprisingly, in the context of cancer, human and mouse Tregs engage in fatty acid synthesis (FAS) to support their functional maturation (32, 33) (**Figure 1**).

FAS is an ATP-consuming process and relies on the rate limiting enzyme acetyl CoA carboxylase (ACC) (**Figure 1**). Inhibition of this rate-limiting step increases FAO and preferentially skews naïve CD4^+^ T cells towards Tregs, delaying disease onset in experimental models of autoimmunity (34). Directing cells to engage in FAO, by replacing glucose with galactose, enhances expression of FoxP3 in Tregs (30). Mitochondrial activity and oxidation of fats are supported in Tregs by fatty acid uptake (35). Fatty acids are characterized by their carbon length – less than 4C are short chain FA (SCFA), medium chain (MCFA) and greater than 12C are long chain FA (LCFA). LCFAs require carnitine palmitoyl transferase 1 (CPT1) to ease mitochondrial transportation by attaching carnitine (e.g. palmitoyl-carnitine), while SCFAs and MCFAs move freely across the membrane (**Figure 1**).

CPT1a loss, the predominant isoform in lymphocytes, inhibits oxidation of LCFA in Tregs. However, this does not affect the development of tissue resident Tregs, *de novo* polarization, or suppression, suggesting that Tregs most likely depend on SCFA and MCFA for FAO (23, 36). Nevertheless, this does not mean that LCFAs do not modulate Treg metabolism. The fatty acid binding protein 5 (FABP5), the dominant isoform in Tregs, binds to LCFAs and negatively regulates Treg function, suggesting an unexpected role for LCFAs in Tregs (37) **(** **Figure 1**).

Tregs more often rewire their metabolism towards mitochondrial oxidative metabolism and rely less on glycolysis, which is in part mediated through the transcriptional activity of FoxP3 (32, 38, 39). It is important to note that glucose is absolutely required for Treg generation and that Tregs transcriptionally express the glucose transporters GLUT1,3,6, and 8. While GLUT1 is expressed by Tregs, it is dispensable for differentiation, suggesting a role for other isoforms (**Figure 1**) (25, 40). In addition, iTregs increase their glycolytic rate, albeit at a lower extent than other CD4 lineages. Tregs generally prefer oxidative glycolysis, which is characterized by glucose being broken down into pyruvate, rather than anaerobic glycolysis where pyruvate is shunted towards lactate production (38, 41) (**Figure 1**).

A common pharmacological approach for disrupting glycolysis is with 2-deoxyglucose (2-DG) which inhibits both phosphoglucose isomerase (PGI) and hexokinases (HK) (42, 43). Tregs express HKI and HKII and 2-DG exposure promotes their differentiation and function by limiting glycolysis (44–46). Inhibition of PGI by 2-DG leads to diversion of glucose 6-phosphate into the pentose phosphate pathway (PPP). Products of the PPP are NADPH, ribose for ribonucleotides and glycolytic intermediates (**Figure 1**) (47, 48). Although the PPP has yet to be investigated in Tregs, 2-DG data suggests that it may play an important role.

Another branching pathway from glycolysis is the hexosamine pathway where fructose-6-phosphate produces glucosamines, primarily UDP-GlcNAC (**Figure 1**). These sugar amines play a critical role in maintaining FoxP3 stabilization and suppressive function through post-translational modifications (49–51).

It is worthy to note that Tregs are not restricted in their ability to engage in aerobic glycolysis. In fact, proliferating and expanding Tregs in response to multiple receptor stimuli, such as Toll Like Receptors (TLR), TCR and CD28, metabolically switch towards aerobic glycolysis (**Figure 1**). In many cases, increasing aerobic glycolysis does not weaken their suppressive activity; on the contrary, glycolysis reinforces suppressive function in human and mouse Tregs (30, 35, 45, 52–55). Interestingly, the glycolytic enzyme, Enolase 1 (ENO1) has a secondary function in human Tregs. ENO1 binds to the *FOXP3* gene as means to regulate *FOXP3* splice forms (De Rosa et al., 2015) (**Figure 1**). Overall, the relationship with glycolysis in Tregs is a complex one. On the one hand its restricted function allows for increased differentiation and on the other hand glycolysis is integral to Treg expansion and suppressive activity.

### Mitochondrial ROS

The function of mitochondria expands beyond that of mere ATP generation. Mitochondria are powerful signalling organelles, including mitochondrial production of reactive oxygen species (mROS) and programmed cell death (56). For example, TCR-activated Tregs couple downstream signalling – namely the T-cell specific tyrosine kinase Lck – with mitochondrial function by mobilizing Lck to the mitochondria (57). Tregs show a clear dependence on mitochondrial activity and as a consequence of increased ETC function, Tregs display enhanced mROS compared to activated effector CD4^+^ T cells (25) (**Figure 1**). Impeding mROS production at complex III (CIII) by genetic deletion of the CIII ROS-producing subunit in Tregs severely reduces suppressive capacity and drives lethal multiorgan inflammation (24). However, mROS can also arise from inhibition of complex I (CI). Inhibition or mutation in CI reduces Treg differentiation and suppressive function *in vivo* (25, 58). Levels of mROS require fine tuning since too much of it becomes damaging to the cell (59). In cases of chronic inflammation, such as autoimmunity, circulating Treg numbers decline and bear hallmarks of damaging mROS leading to cell death (60). Collectively, Tregs are highly dependent on mitochondrial metabolism for their survival, function, and differentiation. Any mitochondrial dysregulation leads to deleterious effects in Tregs.

## Lipids in Transcriptional Regulation

### Peroxisome Proliferator Activated Receptors - PPARs

**Figure 2:**
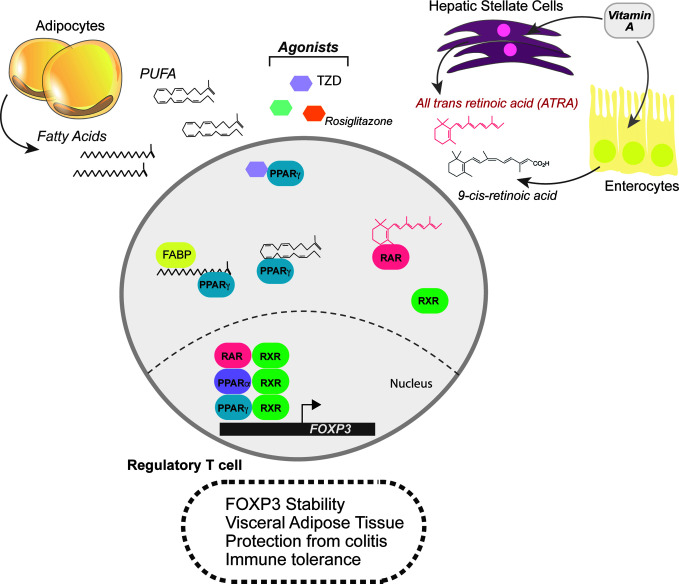
Lipophilic sensors in shaping the transcriptional landscape of Tregs. Fatty acids are also powerful signalling molecules that can change the transcriptional profile of cells. In Tregs, the lipophilic sensors, PPARα, PPARγ, and the retinoic acid receptor (RAR) support Treg differentiation and functional programs. The PPAR family of transcription factors respond to broad range of fatty acids, but quite potently to polyunsaturated fatty acids (PUFAs). Agonists for the PPARs, such as rosiglitazone and thiazolidinedione (TZD) help boost Treg differentiation, FoxP3 stability and function. Visceral Adipose tissue (VAT) Tregs are in constant supply of lipid ligands and upregulate PPARγ. RARα also responds to lipophilic ligands, but only those derived from retinol or retinoic acid. Two key sites of retinoic acid storage and production of all-trans retinoic acid (ATRA) are the liver and enterocytes. In addition, hepatic stellate cells can store retinoids and, when activated, produce ATRA.

Fatty acids and lipid-derivatives can also act as signalling molecules which converge on transcriptional reprograming. The family of peroxisome proliferator activated receptors (PPARs) are part of a super family of nuclear hormone receptors that bind to natural and synthetic lipophilic acids. There are four types of PPARs: PPAR α, β, γ, and δ. Acting as lipid sensors, PPARs heterodimerize with the retinoid receptor RXR within the nucleus and bind to DNA at PPAR response elements (PPRE) (**Figure 2**). For example, endogenous ligands for PPARs are polyunsaturated fatty acids (PUFAs) and eicosanoids that drive transcriptional regulation of glycolytic, lipid and mitochondrial genes (61). The visceral adipose tissue (VAT), an abundant site of lipid metabolism, harbors a distinctive set of Tregs called VAT Tregs that express high levels of PPARγ (**Figure 2**). Indeed, Treg-specific ablation of PPARγ selectively reduces homeostatic levels of VAT Tregs (62). Agonist activation of PPARα and γ with natural and synthetic lipophilic compounds act through transcriptional activity not only to increase iTreg polarization, but also to boost their function *in vitro* and in T-cell mediated colitis (63–65) (**Figure 2**).

Natural PPAR agonists, such as omega-3 fatty acids (Ω-3-FA), which are a class of PUFAs, display immunomodulatory effects (**Figure 2**). Elevating dietary Ω-3-FA or genetically manipulating their levels reduces severity in experimental models of autoimmunity, such as T-cell induced colitis (66–68). In dietary induced obesity, Ω-3-FA supplementation dampens adipose tissue inflammation, in part by promoting *de novo* Treg differentiation (68). In cases of cardiac allografts and milk protein allergies, Ω-3-FA increase peripheral Tregs to achieve immune tolerance (69, 70).

### Retinoic Acid Receptors - RARs

Other nuclear hormone receptors responding to lipophilic metabolites also play key roles in regulating Treg biology. While PPARγ heterodimerizes with RXR, RXR also heterodimerizes with the retinoic acid receptor alpha (RARα). The RAR and RXR family of transcription factors bind to metabolites of retinol (lipid soluble vitamin A), such as all-trans retinoic acid (ATRA) or 9-cis-RA (71). Vitamin A is absorbed in the small intestine and is either catabolized in enterocytes or in the liver (**Figure 2**). ATRA exposure during *de novo* Treg differentiation promotes the expression of gut-homing receptors and RA supplementation increases Tregs at the mucosal interface in experimental models of colitis (72, 73). Pro-inflammatory cytokines weaken Treg stability; however, RA can protect Tregs by preferentially enhancing FoxP3 expression and stabilization (74–79) (**Figure 2**).

Another site of Vitamin A metabolism is the liver. Naïve hepatic stellate cells (HSCs) store retinoids, which can account for nearly 50% of all RA in the body, and produce ATRA upon activation in the local environment (80). In addition to ATRA, activated HSCs can produce TGFβ, a potent combination for Treg differentiation. Co-culture of HSCs with DCs and naïve CD4^+^ T cells skewed differentiation towards Tregs in RARα-dependent manner (81) (**Figure 2**). In conclusion, retinoic acid metabolism, either at mucosal sites or in the liver, can promote an immunosuppressive environment mediated through FoxP3 induction and stabilization.

Of note, other essential vitamins can influence Treg biology. It has been shown that folic acid (Vitamin B9) is necessary for maintenance of Tregs in the colon; mice fed a diet depleted of folic acid were more susceptible to gut inflammation (82). Both vitamin C and D have also been shown to enhance *de novo* Treg induction; however, only vitamin D has demonstrated reduced severity in experimental models of autoimmunity (83–87).

## Oxygen Poor Environments and Treg Adaptation

Tumors have been thought of as “wounds that do not heal”, with many similarities in stromal reorganization reminiscent of wound healing (88). One of the hallmarks of cancer is tissue reorganization which leads to changes in local supply of nutrients and oxygen surrounding the cells (89, 90). One prominent cell type found in cancer are Tregs, which adapt to these metabolically competitive environments.

Changes in oxygen levels within tissues are sensed intracellularly by prolyl hydroxylases (PHD). When oxygen levels are within the normoxic range (20% in tissue culture and around 5% in tissues), PHDs hydroxylate the hypoxia inducible factor 1 α (HIF1α) and target it for proteasomal degradation (91, 92). Under low oxygen levels, HIF1α is released from PHD-dependent degradation and heterodimerizes with HIF1β to translocate to the nucleus. HIF1α upregulates genes involved in aerobic glycolysis, angiogenesis, erythropoiesis, and cell survival as a mechanism for cellular adaptation under hypoxia (93).

Treg migration and survival in inflamed peripheral tissues and tumors depend on metabolic adaptation to support these processes. One major metabolic change in migrating Tregs is their switch towards aerobic glycolysis, which is mediated by glucokinase (GCK) and HIF1α (53, 94). Once within the tissue, Tregs are exposed and adapt to different O_2_ environments. Localized tissue hypoxia can induce the differentiation of Tregs through the direct upregulation of FoxP3 by HIF1α (95). Hypoxia-induced Tregs may help limit tissue damage under states of hypoxic inflammation. The gut has been documented to function under a physiologically hypoxic environment in comparison to other tissues (96). In fact, HIF1α-expressing Tregs repress T-cell induced colitis, suggesting a key role for hypoxia and aerobic glycolysis in mitigating inflammation in oxygen-poor tissues (95). However, under conditions of normoxia, HIF1α negatively regulates Treg differentiation, suggesting that oxygen availability shapes Treg fate (44).

Tumor oxygenation is generally much lower than that of normal tissues, yet Tregs readily populate tumors to provide additional immune tolerance to support tumor growth (97, 98). For instance, the brain is highly sensitive to oxygen variability and functions around 4.4%pO_2_, yet Tregs manage to adapt in order to survive under oxygen competitive environments (99, 100). Tregs in glioma use HIF1α to support this metabolic adaptation by increasing aerobic glycolysis for migration and depend mainly on fatty acid uptake to support OXPHOS-mediated immune suppression (94, 101). However, other tissues have different levels of O_2_ supply and degrees of O_2_ sensitivity. Oxygen ranges from 5.6-14.5% pO_2_ in the lung (100) suggest that Tregs may adapt accordingly. In the case of lung metastasis, PHD, the negative regulator of HIF1α is activated in Tregs and drives their accumulation in the lung to create an immune-tolerant metastatic niche (102).

**Figure 3:**
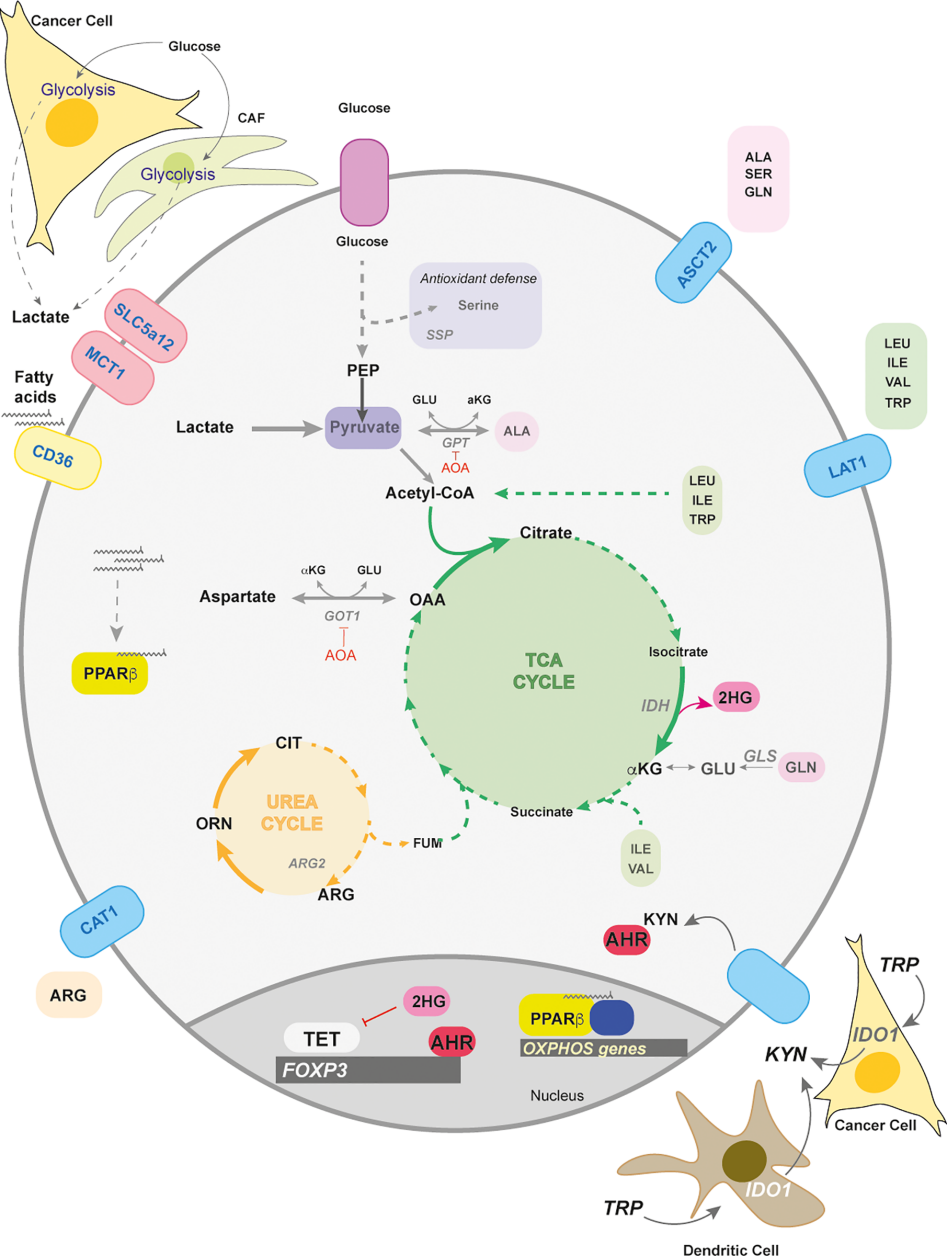
Lactate and amino acid metabolism in Tregs. Lactate and amino acid metabolism in Tregs play important roles in their differentiation and function. Lactate can arise in the tumors and from cancer associated fibroblasts (CAFs) as a consequence of their highly glycolytic nature. The transporters for lactate are SLC5a12 and MCT1. Tregs metabolically adapt to a low glucose/high lactate environment by using lactate to fuel the TCA cycle that supports oxidative phosphorylation in the mitochondria. Intra-tumoral Tregs increase the fatty acid receptor CD36 when extracellular lactate concentrations increase. The import of fatty acids activates the transcription factor PPARβ to maintain mitochondrial fitness. Other transported nutrients are amino acids. ASCT2, a neutral amino acid transporter, is responsible for the intake of amino acids such as alanine, serine, glutamine. Serine uptake and biosynthesis (serine synthesis pathway – SSP) are involved in antioxidant defence in Tregs. The amino acids, alanine and aspartate, can enter central carbon metabolism through transamination by glutamate pyruvate transaminase (GPT) and glutamate oxaloacetate transaminase (GOT), both of which are negative regulators of FoxP3 induction. Both GOT and GPT are inhibited by the transaminase inhibitor, aminooxyacetate (AOA). Another negative regulator is alpha-ketoglutarate (αKG) which can be converted by the error-prone isocitrate dehydrogenase (IDH) to 2-hydroxyglutarate (2-HG). 2-HG inhibits the family of DNA demethylases – ten eleven translocase (TET) 1-3. Another amino acid that can enter the TCA cycle is arginine which is transported by the cationic transporter (CAT1). Arginine helps fuel the urea cycle, where arginase (ARG2) converts arginine to ornithine and later citrulline. The transport of branched chain amino acids (BCAA), and aromatic amino acids, such as isoleucine and tryptophan, use the L-type amino acid transporter (LAT1). BCAA can help fuel the TCA either through acetyl CoA or succinate generation. Tryptophan (TRP) can be converted into kynurenine by the Indoleamine 2,3-dioxygenase (IDO1) in dendritic or tumor cells, which activates the ligand receptor, Aryl hydrocarbon Receptor (AhR). Ligand-bound AhR translocates to the nucleus and upregulates *Foxp3* expression.

In response to low O_2_ levels, the acidity of the local milieu within tumors increases compared to normal tissue (103). This increase in acidity imposes harsh conditions for surrounding T cells. One main metabolite that could regulate local acidity is lactate; the major transporters for lactate in T cells are SLC5a12 and MCT1 (41, 104–106) (**Figure 3**). Tregs within the tumor interstitial space are able to outcompete other T cell subsets by surviving under low glucose and high lactate conditions. Intratumoral Tregs upregulate the SLC5a12 and MCT1 allowing lactate to fuel the TCA-cycle for OXPHOS-mediated immune suppression and to support gluconeogenic pathways for survival (41, 106) (**Figure 3**). Limiting tumor-derived lactate in the context of anti-CTLA4 blockade increases glucose consumption in intratumoral Tregs and skews the balance towards IFN-γ producing CD4^+^ T cells (107).

Other stromal components, such as cancer associated fibroblasts (CAF), contribute to lactate production that preferentially selects for intratumoral CD4^+^ Foxp3^+^ populations through a NF-kB-FoxP3 axis (108) (**Figure 3**). Another method of survival under lactic acid rich environments is achieved by upregulating the fatty acid receptor CD36. Intratumoral Tregs particularly express CD36 in order to enhance PPARβ expression as a means to support mitochondrial metabolism in Tregs and their survival (109) (**Figure 3**). High levels of environing lactate due to the highly glycolytic nature of cancer cells and CAFs could be part of a strategy employed by tumors to promote immune suppression.

## Tissues and Amino Acid Metabolism in Tregs

Other immunomodulatory nutrients found in inflammatory sites and tumor lesions are amino acids and their catabolic by-products (110, 111). General amino acid uptake and breakdown is imperative for T cell proliferation and CD4^+^ T_helper_ differentiation. Known amino acid (AA) transporters expressed on the surface of T cells are the SLC7A5/SLC3A2 (LAT1), and SLC1A5 (ACST2) (112, 113).

### Non-Essential Amino Acids

#### Glutamine

Typical amino acids transported by ASCT2 are alanine, serine, cysteine, methionine and glutamine (114). Interestingly, ASCT2 is dispensable for thymic development of nTregs and *de novo* polarization (112). However, glutamine deprivation increases FoxP3 expression upon TCR engagement and also increases in a TGFβ-dependent manner (115, 116). Glutamine restriction has also been shown to enhance suppressive function in a T-cell mediated colitis model (115), which could also be a method for tumors to promote immune suppression (**Figure 3**).

How glutamine negatively regulates Treg induction and activity is still poorly understood. Interestingly, deletion of glutaminase (GLS), an amidohydrolase enzyme that converts glutamine to glutamate, does not affect homeostatic Treg frequencies nor their differentiation *in vitro* (117) (**Figure 3**). This would suggest that Tregs do not depend on glutamine to fuel the TCA cycle and further supports the notion that Tregs utilize other carbon sources, such as fatty acids (**Figure 3**).

Unexpectedly, increased transamination - the transfer of amine groups either from glutamine or glutamate that generates glutamate or αKG respectively – negatively impacts Treg biology. Aminooxyacetate (AOA), a global transaminase inhibitor, increases FoxP3 expression both in Treg and Th17 polarization conditions (118) (**Figure 3**). Deletion of GOT1, which transfers amino groups between aspartate and glutamate interchangeably to produce OAA and αKG, rewires Th17 cells towards Tregs *in vitro* and in experimental autoimmune encephalomyelitis (EAE). This effect was partially due to the promiscuous nature of dehydrogenases (e.g. isocitrate dehydrogenase – IDH) leading to conversion of αKG into 2-hydroxyglutarate (2-HG). Elevated levels of 2-HG inhibit the DNA demethylating enzymes TET1-3 and this inhibition keeps the *Foxp3* promoter silenced (118) (**Figure 3**).

#### Arginine

While the majority of amino acids are neutral, basic amino acids, such as arginine and to a lesser extent histidine, require cationic amino acid transporters (CAT) (119). CAT1 is highly expressed on proliferating T cells and transports arginine downstream of TCR engagement (120) (**Figure 3**). In cases of muscle wound healing, a specific Treg population infiltrates the site for damage resolution as well as causes a local increase in arginine metabolism (110, 121). Intracellular arginine can be broken down in the urea cycle by arginase (ARG1 and ARG2) into ornithine and citrulline (122). Activated Tregs found in the skin enhance ARG2 expression to outcompete and deprive effector T cells of environmental arginine (123) (**Figure 3**). Tregs can also utilize citrulline, a component of the urea cycle, for enhancing FoxP3 induction, thus potentially still surviving in arginine competitive environments, such as in arginine-addicted tumors (124, 125) (**Figure 3**).

#### Serine

Serine is a non-essential amino acid implicated in numerous processes such as glycine synthesis, purine synthesis, anti-oxidant defence and the methylation cycle whose overall function is critical for T effector cell function (126). Anti-oxidant programs in T cells include the *de novo* generation of glutathione from glutamate, cysteine and glycine by glutamate cysteine ligase (Gclc) (127). Gclc-loss in Tregs drives ROS-dependent serine uptake and *de novo* serine synthesis leading to multiorgan autoimmunity (128). Impeding serine metabolism in Gclc-null Tregs rescues Treg suppressive activity, suggesting that reduced glutathione is crucial for Treg function by restraining serine metabolism (**Figure 3**).

### Essential Amino Acids

Essential amino acids also require transporters for uptake; for example, LAT1 imports branched chain amino acids (BCAA) and aromatic amino acids (e.g. Phenylalanine, histidine, leucine and tryptophan) at the expense of exporting glutamine (129). Interestingly, BCAA bypass first-pass hepatic metabolism, thus allowing for the muscle (primarily) and other organs to immediately acquire BCAA (130). In addition to being essential to protein synthesis, BCAA help replenish the TCA cycle and provide carbon units for fatty acids synthesis (131). Disrupting BCAA metabolism by limiting dietary isoleucine diminishes homeostatic numbers of Tregs and weakens their suppressive function. Disrupting part of the BCAA transporter, SLC3A2, similarly reduces Treg homeostatic survival and their suppressive activity in experimental colitis models (132). In some cases of cancer, such as pancreatic cancer, there is an early rise of serum BCAA (133), which may perhaps support Treg function in limiting anti-tumor immunity, but this is yet to be explored.

#### Tryptophan and Kynurenine

One well described mechanism of tumor evasion is the expression by cancer cells or tolerogenic DCs of tryptophan-degrading enzymes such as indoleamine-pyrrole 2,3-dioxygenase 1 (IDO1) (**Figure 3**). This enzyme catalyzes the conversion of tryptophan into kynurenine in a pathway that leads to the production of kynurenic acid, quinolinic acid and NAD. Both the reduced presence of tryptophan and the accumulation of kynurenine have been shown to potentiate an immunosuppressive cancer microenvironment (134–136).

One IDO-dependent mechanism of promoting immune suppression is the generation of Tregs. It has been shown that under low-tryptophan/high kynurenine or IDO1-expressing DC’s, naïve CD4^+^ T cells enhance FoxP3 expression (137, 138) (**Figure 3**). In a mouse model of B16-OVA tumor cells implanted in FoxP3-GFP reporter mice, IDO1 inhibition led to increased IL-17 expression in Tregs cells suggesting that tryptophan and kynurenine metabolism play an important role in Th17/Treg plasticity *in vivo* (139).

### Intracellular Sensing of Amino Acids by mTOR

Amino acids play a critical role in modulating Treg homeostasis, polarization, and function; however, T cells require intracellular nitrogen sensors to couple availability with function. One major nitrogen sensor is the mammalian target of rapamycin (mTOR) Ser/Thr kinase. mTOR assembles into two distinct complexes termed complex 1 (mTORC1) and complex 2 (mTORC2), with mTOR harboring the catalytic function for both complexes. Amino acids, growth factors, and glucose can stimulate mTORC1 activation for global protein synthesis (140). Translation consumes around 25% of cellular ATP in lymphocytes and requires charged amino acids as building blocks for protein synthesis (141), thus providing mTORC1 with the responsibility of sensing amino acid availability to keep translation in check.

Amino acids promote the Rag GTPases to recruit mTORC1 to the lysosomal membrane and bring mTORC1 in close proximity to the small GTPase Rheb (142). GTP-bound Rheb then activates mTORC1 (143). The many functions of mTORC1 include downstream targets involved in protein synthesis (e.g. S6 ribosomal protein) and lipid synthesis (144). Loss of the mTOR catalytic subunit in T cells and Treg-specific loss increases FoxP3 induction, in part mediated by the mTORC2 complex. However, mTOR catalytic activity and mTORC1 are indispensable for suppression by regulating transcripts involved in mitochondrial oxidative and lipid metabolism (28, 145–148). Foxp3-specific ablation of upstream mTORC1 activators, RagA/B and Rheb, severely impair Treg suppressive function leading not only to uncontrolled systemic inflammation, but also enhanced anti-tumor immunity (147–149). Interestingly, activation of human Tregs has identified an oscillatory role for mTORC1 activity in controlling metabolic plasticity (150).

Amino acids are essential for T cell proliferation and Tregs are no exception. While glutamine and serine metabolic pathways are critical to T effector cells, they seem to reduce Treg differentiation and activity. Tregs rely on BCAA and arginine metabolism potentially as means to support the TCA cycle for OXPHOS. The activation of the amino acid sensor, mTORC1, is essential for the suppressive fitness of Tregs. These data collectively suggest that amino acid metabolism and sensing are key regulators of Treg homeostasis, lineage polarization, and function.

## The Gut and Microbiome Metabolite By-Products

**Figure 4:**
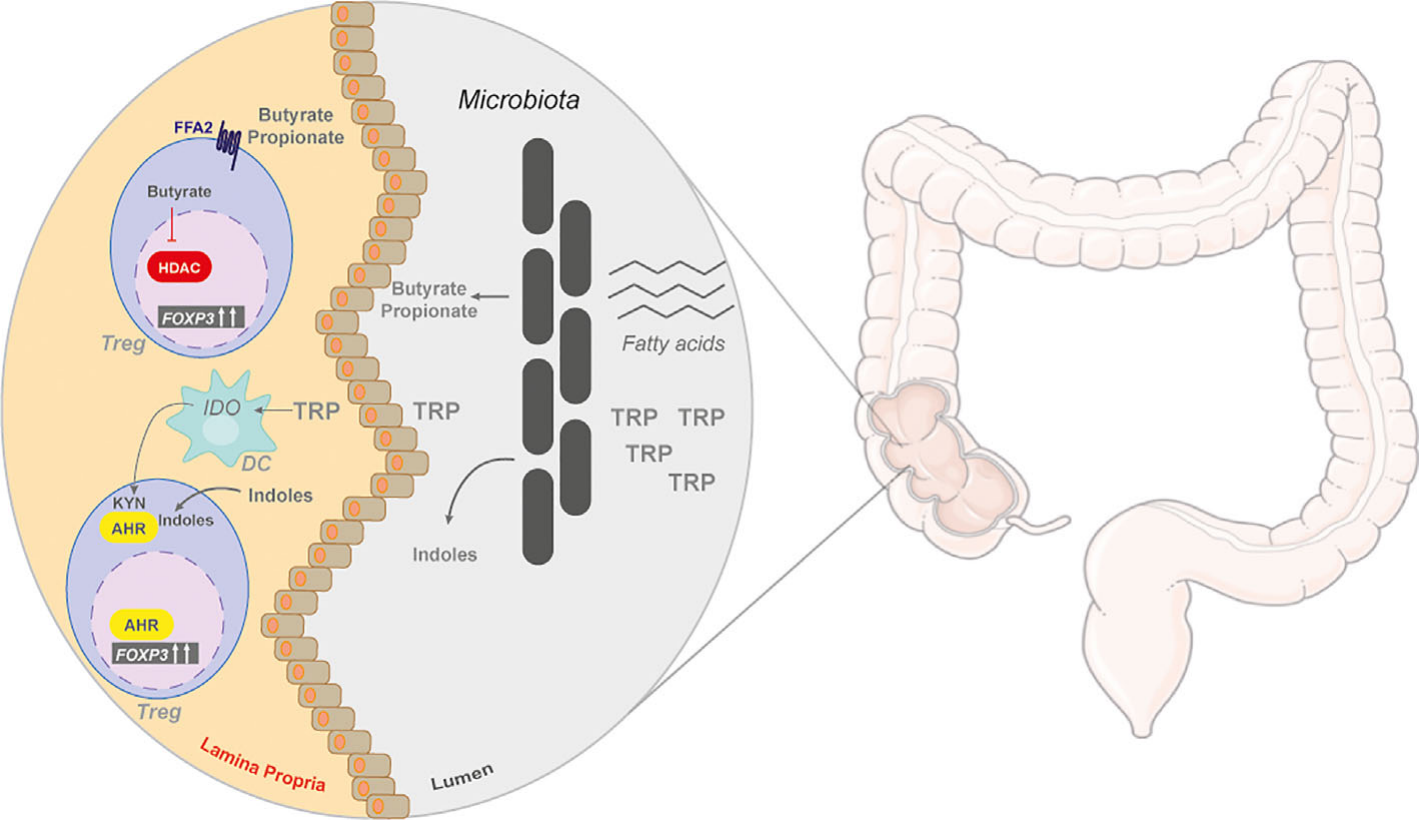
The gut and microbiome by-products in regulating Tregs. The colon is a metabolically active site, with many unabsorbed nutrients, such as amino acids and fats. These nutrients are metabolized by the gut commensal bacteria. Unabsorbed fatty acids can be converted to short chain fatty acids (SCFAs), butyrate and propionate, that are released and enter the lamina propria. In the colonic lamina propria, SCFAs can induce the differentiation of naïve T cells into iTregs through the G protein-coupled receptor (GPCR), free fatty acid receptor 2 (FFA2), or through its inhibitory action on histone deacetylases (HDACs). Tryptophan (TRP) is also catabolized by gut symbionts into indoles or can be catabolized by dendritic cells (DC) into kynurenine (KYN) by indoleamine-pyrrole 2,3-dioxygenase 1 (IDO), both of metabolites activate AhR.

The colon is an extraordinary organ of the mammalian body, as it holds a large mass of bacterial species. It has been documented that 10-20% of faecal weight is contributed by bacteria and >90% are obligate anaerobes (151). Most importantly, these anaerobic bacteria further metabolize unabsorbed peptides, fats and oligosaccharides, reaching a metabolic activity equivalent to that of the liver (152, 153) (**Figure 4**). The by-products of anaerobic fermentation in the gut are organic amines from peptides, ammonia from peptides and nitrogen-based metabolites, and SCFAs (i.e. acetate, butyrate, and propionate) from dietary fibre (153). SCFAs are water soluble and allow for easy absorption. It has been observed since the 1970’s that germ-free mice suffer from chronic diarrhoea, which could be remedied by providing SCFAs, suggesting that bacterial SCFAs are responsible for maintaining colonic homeostasis (154)

Tregs are a key modulator of colonic homeostasis with luminal concentrations of SCFAs positively correlating with colonic Treg frequencies (155) (**Figure 4**). Germ-free mice have reduced colonic Tregs that can be rescued by butyrate supplementation, suggesting that bacterial fermentation of dietary fibre into SCFAs helps maintain colonic Treg pools (156). Butyrate, and to a lesser extent propionate, can induce Tregs in a TGFβ-dependent manner *in vitro* and in models of T-cell-induced colitis (155, 156). Since SCFAs are water soluble, Treg accumulation in other organs can also occur, providing beneficial outcomes in experimental models of type I diabetes and kidney allograft transplantation (157, 158).

SCFAs can act in two ways to promote Treg accumulation: G protein-coupled receptor (GPCR) and histone deacetylases (HDAC) (**Figure 4**). Loss of the free fatty acid receptor, FFA2, obliterates any Treg-inducing effect of SCFAs in the colon and in allograft transplantation, suggesting that signalling through the GPCR regulates FoxP3 induction (156, 157). Butyrate treatment also increases histone H3 acetylation on the promoter of the FoxP3 locus by inhibiting class I and class II HDACs (155, 159, 160). These two mechanisms employed by SCFAs are perhaps linked, but this has yet to be investigated.

Another abundant dietary nutrient catabolized by the gut and microbiome is tryptophan, an aromatic amino acid with an indole side chain. Tryptophan can be broken down by both the host and commensal bacteria in the gut. Host degradation of tryptophan is used as a precursor to serotonins and niacin (vitamin B3) biosynthesis (161). A few commensal bacterial species in the gut, such as *Escherichia coli*, metabolize tryptophan and release indoles and indole-derivatives into circulation (162) (**Figure 4**). Dietary administration of indoles, such as 2-(1*H*-Indol-3-ylcarbonyl)-4-thiazolecarboxylic acid methyl ester (ITE), protect against experimental models of colitis, EAE, and type I diabetes (159, 163–166). Indoles and their derivatives were found to activate the Aryl hydrocarbon receptor (AhR). AhR is a ligand-activated transcription factor with a promiscuous binding site recognizing synthetic and natural ligands (167, 168). Indole-derived ligands enhance TGFβ-induced Treg differentiation and increase IL10 production in activated T cells *in vitro* (163, 169) (**Figure 4**). Dietary indole supplementation promotes Treg frequencies in mice by directly activating AhR or by modifying the microbiota towards butyrate-producing species (170, 171). However, it should be noted that not all protective qualities of indoles act through enhancing Treg activity in models of autoimmunity (165).

The intestinal tissue also contributes to ligand activation of AhR through the degradation of tryptophan to NAD by the kynurenine pathway (167). The by-products of this pathway (e.g. kynurenine) activate AhR (167) (**Figure 3**). Other sources of kynurenine are gut-specific CD11c^+^CD103^+^ DCs expressing IDO (**Figure 4**). This DC subset enhances Treg differentiation as a means to mediate host-microbe homeostasis in the gut (172). Overall, tryptophan metabolism either through host or microbial breakdown can have a profound impact on Treg homeostasis and differentiation in the host.

## Conclusion

Nutrient availability and the production of various intracellular metabolites can shape Treg cell fate and function (**Table 1**). The key metabolic cues governing Tregs are driven by mitochondrial oxidative metabolism, mainly the TCA cycle and FAO, along with support from glycolytic intermediates. However, in the process of trafficking within the host and reaching new tissue sites, Tregs quickly adapt to new carbon sources and oxygen levels through metabolic rewiring in accordance with the microenvironment. While tissue resident Tregs have been identified in nearly every organ, the type of metabolism that is required for them to survive in these environments is only starting to be understood.

**Table 1:** Metabolites in shaping Treg biology. Tregs are sensitive to various metabolites. The presence of certain metabolites can increase Treg differentiation and suppressive function. However, in some cases, as with 2-hydroxyglutarate, limit Treg differentiation.

Metabolite	Pathway	Effect on Tregs
Glucose	Glycolysis and branching pathways	↑Treg differentiation ↑Proliferation
Acetyl-CoA increase	Oxidative metabolism	↑Treg differentiation
Fatty acids	FAO	↑Treg differentiation
UDP-GlcNAC	Hexosamine pathway	↑Treg Differentiation ↑FoxP3 stabilization ↑Suppressive Activity
W3 fatty acids	Polyunsaturated Fatty Acids (PUFAs)	↑Treg differentiation
Retinoic acid	Vitamin A metabolism	↑Treg differentiation ↑FoxP3 stabilization ↑Suppressive activity
Folic acid	Vitamin B9 metabolism	↑Treg maintenance
Vit B	Dietary vitamins	↑Treg differentiation
Vit C	Dietary vitamins	↑Treg differentiation
Lactate	Tricarboxylic acid cycle	↑Treg function
Glutamine restriction	Tricarboxylic acid cycle Amino acids	↑Treg differentiation ↑Suppressive activity
2-Hydroxyglutarate	Epigenetics	↑*Foxp3* silencing ↓Treg differentiation
Citrulline	Urea cycle	↑Treg differentiation
Serine	One carbon metabolism	Treg maintenance
Isoleucine restriction	Branched chain amino acids	↓Treg maintenance ↓Suppressive activity
Kynurenine	Tryptophan metabolism	↑Treg differentiation
Butyrate, propionate, acetate (Microbiome derived)	Short chain fatty acids from anaerobic fermentation	↑Treg differentiation ↑Suppressive activity
Indole derivatives (microbiome derived)	Tryptophan metabolism	↑Treg differentiation

In recent years, the democratisation of single cell RNA sequencing has led to numerous studies aiming to understand the transcriptome of Tregs and their potential metabolic landscape (173, 174). However, cellular metabolism is per definition a dynamic process that cannot be fully encapsulated by transcriptomics. In fact, further proteomic studies of Tregs would greatly benefit the field of Treg immunometabolism (175). While 2-NBDG, a fluorescent analogue of 2-DG, has helped in the field of immunometabolism and tissue Tregs to measure glucose uptake, a recent study has suggested it as an unreliable tool as it retains intracellular transport in the presence of multiple GLUT inhibitors (176). The most promising technology available to help disentangle the complex metabolic interface between Tregs and tissues is mass spectrometry imaging. Techniques such as MALDI or DESI-MSI in combination with classical immunohistochemistry confers a spatial-temporal resolution which would provide a more accurate picture of Treg metabolism within tissues (177).

On a final note, our understanding of Treg metabolism has mainly been a blend of findings from both mice and humans. However, it is important to underscore that human Tregs are distinct from their mouse counterparts both metabolically and phenotypically (178, 179). For example, one metabolic difference is that *ex-vivo* human Tregs are glycolytic in nature, while *ex-vivo* mouse Tregs are not (35, 178). A feature that may explain some of the metabolic differences between human and mouse Tregs could lie in the functional heterogeneity of circulating human Tregs – an aspect that is absent in mice (179). While mouse FoxP3^+^ Tregs show functional homogeneity, human FOXP3^+^ Tregs are a pool of Tregs with various phenotypes and different levels of suppressive activity (180). These phenotypic differences will inevitably complicate the understanding of the metabolic landscape of human Tregs and underline a limitation of mouse-to-human Treg metabolism translation.

The nutrient environment in each tissue – be it mouse or human- is a complex formula, yet Tregs find ways to metabolically adapt to these new environments. By understanding these nodes of adaptation, new therapies can emerge to either promote their function in autoimmunity or limit their efficacy in cancer.

## Author Contributions

JB, MH, and FZ conceptualized the review. JB and MH wrote the review with significant input from FZ. All authors contributed to the article and approved the submitted version.

## Funding

This work was funded by Cancer Research UK grant C596/A26855 and supported by the Francis Crick Institute which receives its core funding from Cancer Research UK (FC0010557), the UK Medical Research Council (FC0010557) and the Wellcome Trust (FC0010557).

## Conflict of Interest

The authors declare that the research was conducted in the absence of any commercial or financial relationships that could be construed as a potential conflict of interest.
